# Supplementary material for: The Association between Prenatal Per- and Polyfluoroalkyl Substances Exposure and Neurobehavioral Problems in Offspring: A Meta-Analysis
Source: Int J Environ Res Public Health. 2023 Jan 17;20(3):1668. doi: 10.3390/ijerph20031668 (PMC9914055; doi:10.3390/ijerph20031668)
Supplement: Supplementary file 1 [file ijerph-20-01668-s001.zip › ijerph-2116962-supplementary.pdf]

Table S1. Quality of case-control studies included in meta-analysis according to the Newcastle-Ottawa Scale

Study	Adequate definition of the cases	Representativeness of the cases	Selection of the controls	Definition of the controls	Comparability of cases and controls on the basis of the design or analysis	Ascertainment of exposure	Same method of ascertainment for cases and controls	Non-response rate	Total scores
Zeyan Liew	1	1	1	1	1	1	1	0	7
Amanda Ode	1	1	1	1	2	1	1	1	9
Birgit Bjerre Høyer	0	1	1	1	2	1	1	0	7
Ann M. Vuong	1	1	1	1	2	1	1	0	8
Kristen Lyall	1	1	1	1	2	1	1	0	8
Thea S. Skogheim	1	1	1	1	2	1	1	0	8
Manhai Long	1	1	1	1	2	1	1	1	9

Table S2. Quality of cohort studies included in meta-analysis according to the Newcastle-Ottawa Scale

Study	Representativeness of the exposed cohort	Selection of the nonexposed cohort	Ascertainment of exposure	Demonstration that outcome of interest was not present at start of study	Compare ability of cohorts on the bias of the design of analysis	Assessment of outcome	Was follow-up long enough for outcomes to occur	Adequacy of follow-up cohorts	Total scores
Youssef Oulhote	1	1	1	1	1	1	1	0	7
Marin Strøm	1	1	1	1	1	1	1	1	8
Jiwon Oh	1	1	1	1	1	1	0	1	7
Hyeong-Moo Shin	1	1	1	1	2	1	1	1	9

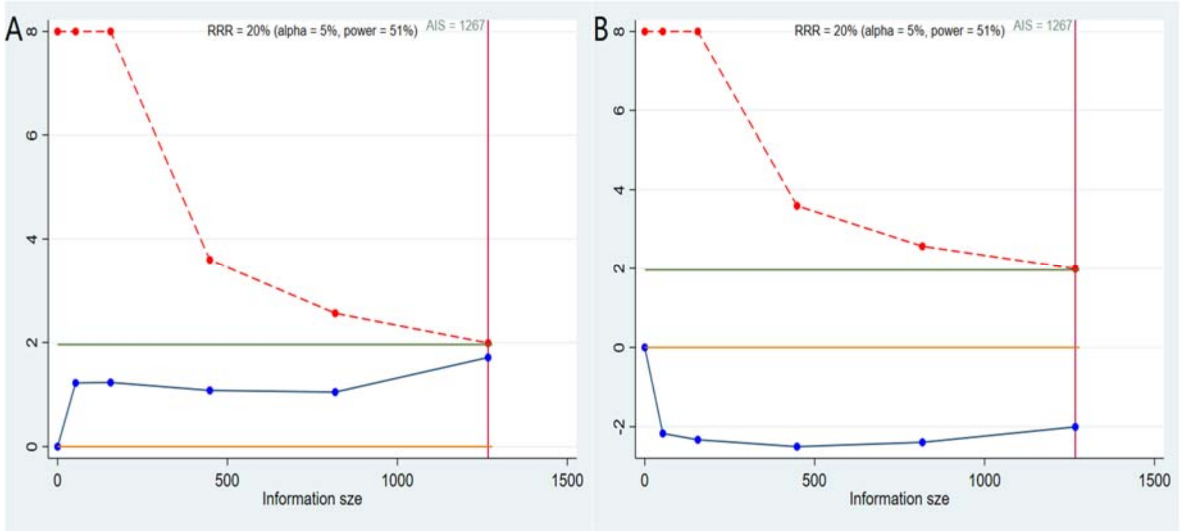

Figure S1. Trial sequential analysis for association between polyfluoroalkyl substances and ADHD (A: PFOA; B: PFOS) Abbreviations: PFOA, perfluorooctanoate; PFOS, perfluorooctane sulfonate; ADHD: attention deficit hyperactivity disorder.

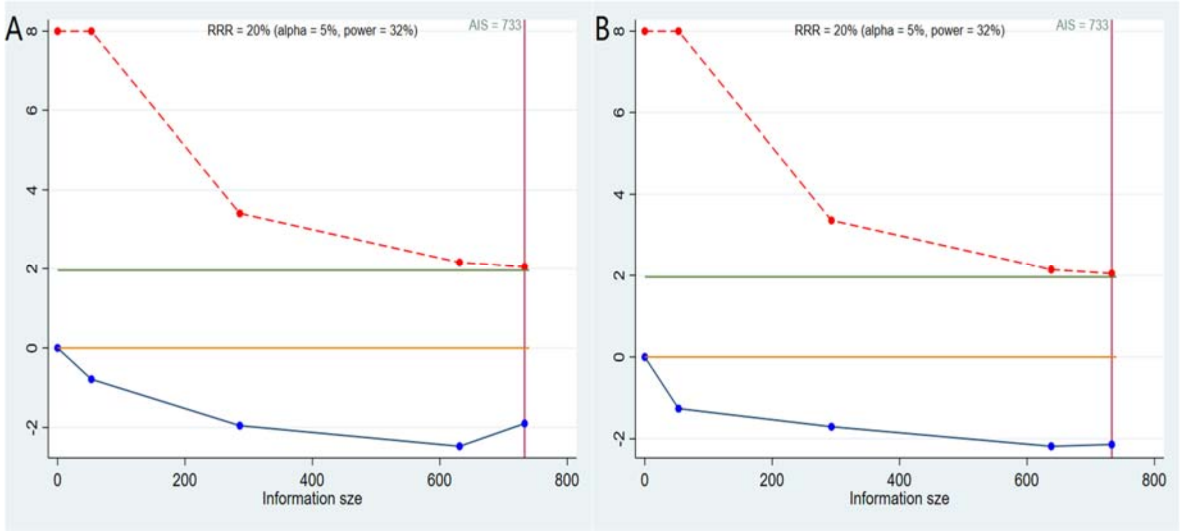

Figure S2. Trial sequential analysis for association between polyfluoroalkyl substances and ASD (A: PFOS; B: PFNA) Abbreviations: PFOS, perfluorooctane sulfonate; PFNA, perfluorononanoate; ASD: autism spectrum disorders.

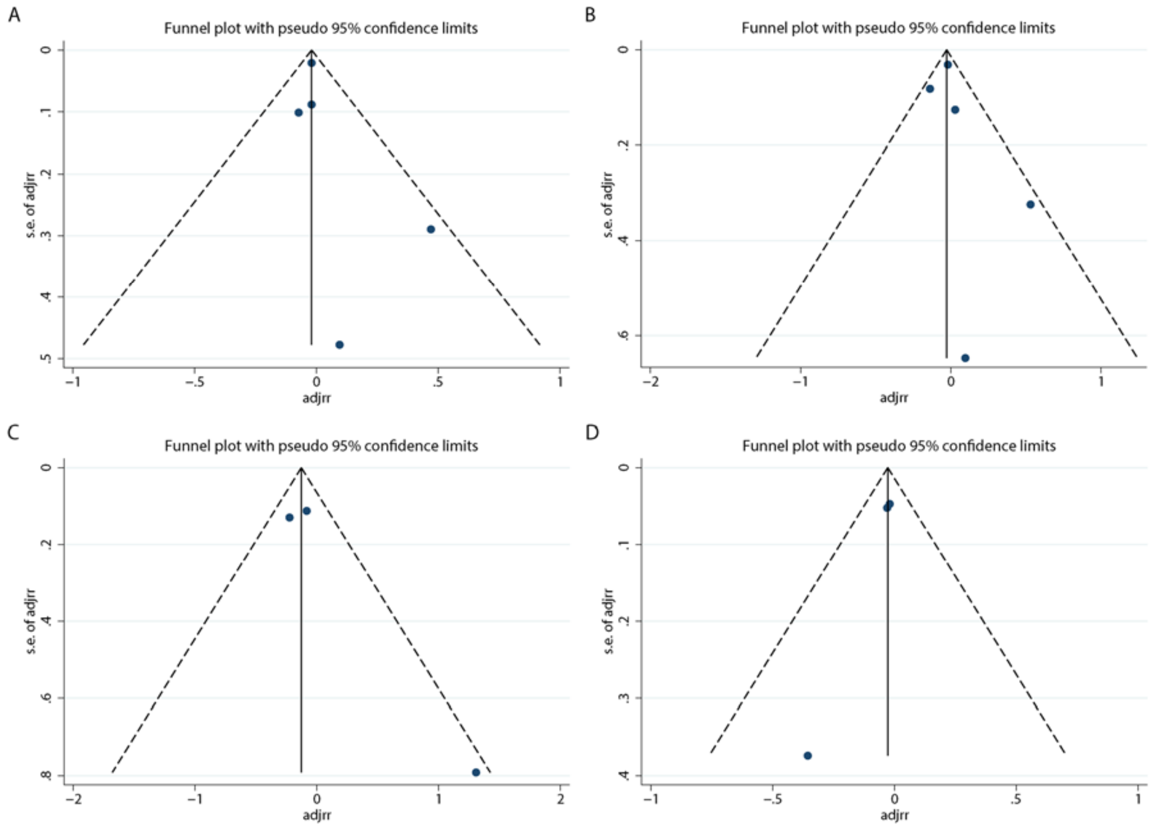

Figure S3. Funnel plot for per 1 ng/ml increase of polyfluoroalkyl substances and ADHD. (A: PFOA; B: PFOS; C: PFNA; D: PFHxS). Abbreviations: CI, confidence interval; PFOA, perfluorooctanoate; PFOS, perfluorooctane sulfonate; PFNA, perfluorononanoate; PFHxS, perfluorohexane sulfonate; ADHD: attention deficit hyperactivity disorder.

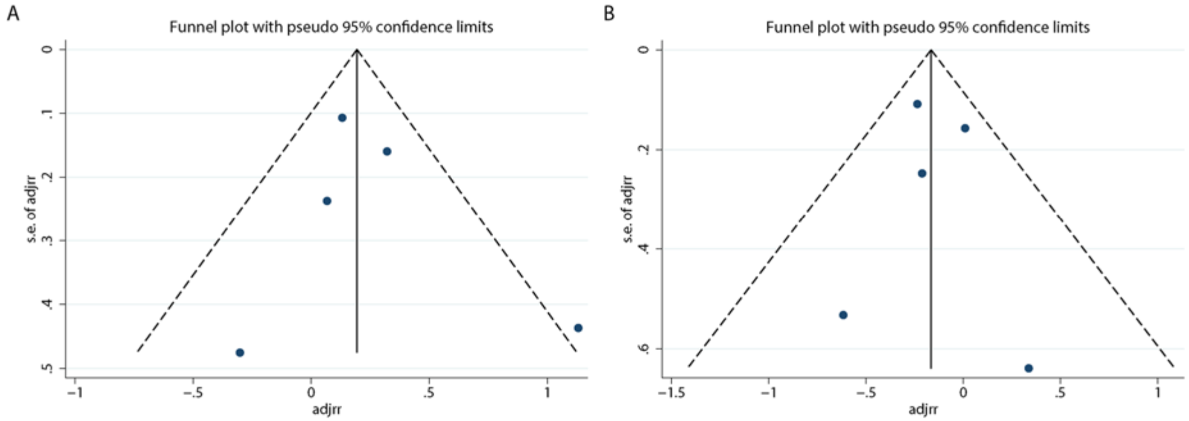

Figure S4. Funnel plot of maximum quartile polyfluoroalkyl substances and ADHD. (A: PFOA; B: PFOS). Abbreviations: Adjrr: adjusted risk ratio; PFOA, perfluorooctanoate; PFOS, perfluorooctane sulfonate; ADHD: attention deficit hyperactivity disorder.

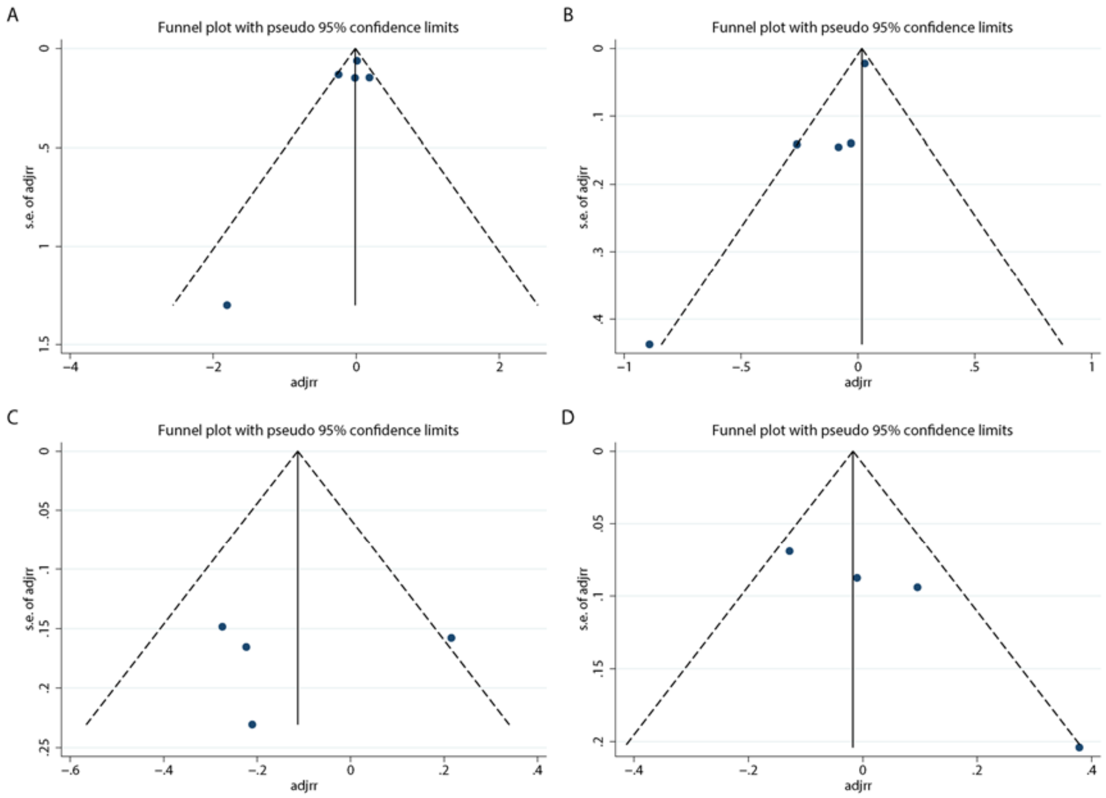

Figure S5. Funnel plot for per 1 ng/mL increase of polyfluoroalkyl substances and ASD. (A: PFOA; B: PFOS; C: PFNA; D: PFHxS). Abbreviations: CI, confidence interval; PFOA, perfluorooctanoate; PFOS, perfluorooctane sulfonate; PFNA, perfluorononanoate; PFHxS, perfluorohexane sulfonate; ASD: autism spectrum disorders.

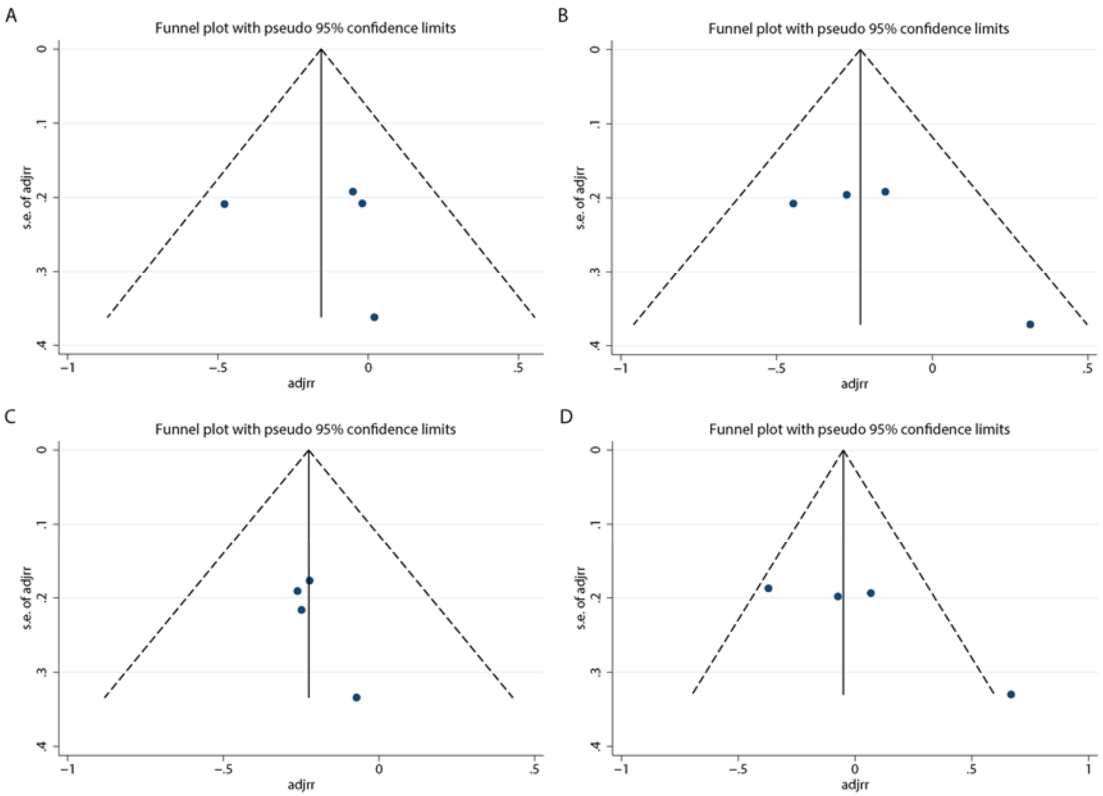

Figure S6. Funnel plot of maximum quartile polyfluoroalkyl substances and ASD. (A: PFOA; B: PFOS; C: PFNA; D: PFHxS). Abbreviations: CI, confidence interval; PFOA, perfluorooctanoate; PFOS, perfluorooctane sulfonate; PFNA, perfluorononanoate; PFHxS, perfluorohexane sulfonate; ASD: autism spectrum disorders.
